# A History of Repeated Alcohol Intoxication Promotes Cognitive Impairment and Gene Expression Signatures of Disease Progression in the 3xTg Mouse Model of Alzheimer’s Disease

**DOI:** 10.1523/ENEURO.0456-22.2023

**Published:** 2023-07-04

**Authors:** Pietro Paolo Sanna, Chiara Cabrelle, Tomoya Kawamura, Daniele Mercatelli, Nathan O'Connor, Amanda J. Roberts, Vez Repunte-Canonigo, Federico M. Giorgi

**Affiliations:** 1 Department of Immunology and Microbiology, The Scripps Research Institute, La Jolla, California 92037; 2 Department of Pharmacy and Biotechnology, University of Bologna, 40126 Bologna, Italy; 3 Research and Development, MBF Bioscience, Williston, Vermont 05495; 4 Animal Models Core, The Scripps Research Institute, La Jolla, California 92037

**Keywords:** transcriptomics, single-cell, Alzheimer's disease AD, alcohol, neuroinflammation

## Abstract

The impact of alcohol abuse on Alzheimer’s disease (AD) is poorly understood. Here, we show that the onset of neurocognitive impairment in a mouse model of AD is hastened by repeated alcohol intoxication through exposure to alcohol vapor, and we provide a comprehensive gene expression dataset of the prefrontal cortex by the single-nucleus RNA sequencing of 113,242 cells. We observed a broad dysregulation of gene expression that involves neuronal excitability, neurodegeneration, and inflammation, including interferon genes. Several genes previously associated with AD in humans by genome-wide association studies were differentially regulated in specific neuronal populations. The gene expression signatures of AD mice with a history of alcohol intoxication showed greater similarity to the signatures of older AD mice with advanced disease and cognitive impairment than did the gene expression signatures of AD mice not exposed to alcohol, suggesting that alcohol promotes transcriptional changes consistent with AD progression. Our gene expression dataset at the single-cell level provides a unique resource for investigations of the molecular bases of the detrimental role of excessive alcohol intake in AD.

## Visual Abstract

**Figure:**
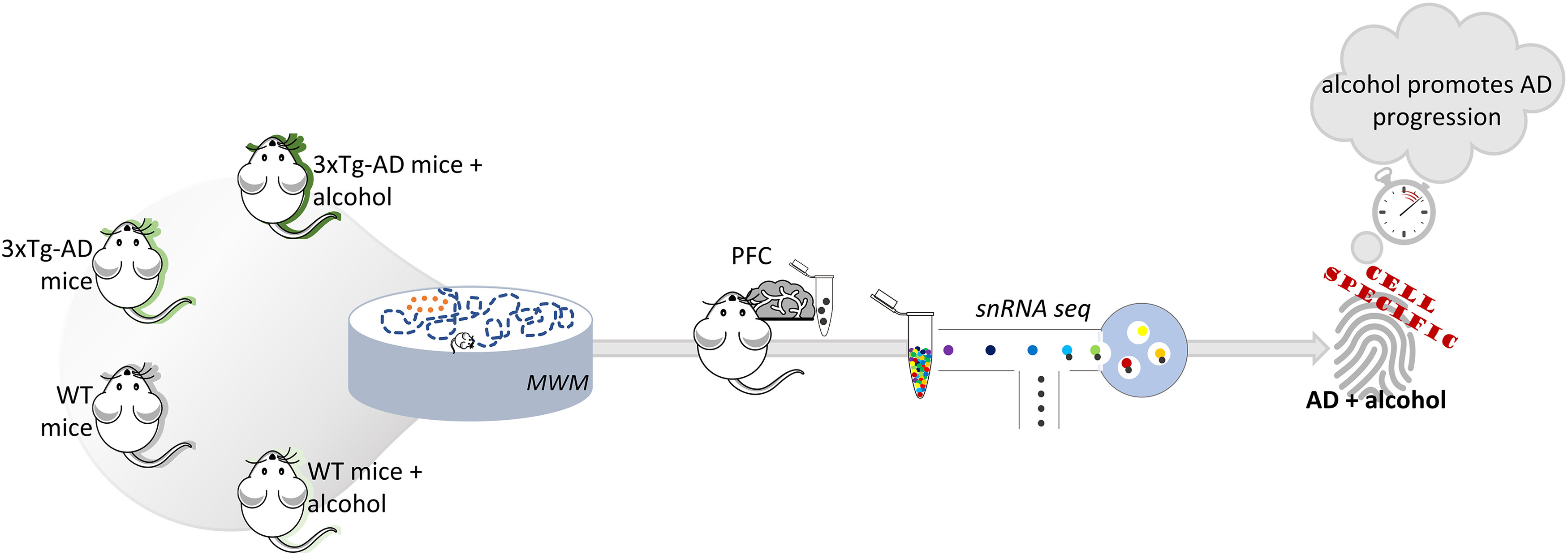

## Significance Statement

Alzheimer’s disease (AD) is the most common neurodegenerative disease worldwide. Many efforts have been geared toward the identification of environmental and genetic risk factors. However, alcohol has received limited attention as a potential risk factor for AD. We explored effects of the interaction of a history of alcohol intoxication with genetic AD susceptibility on cognitive performance and gene expression at the single-cell level. We found that a history of repeated alcohol intoxication promotes the emergence of spatial learning and memory impairments in presymptomatic triple transgenic AD (3xTg-AD) mice. We also show that a history of repeated alcohol intoxication induces prefrontal cortex transcriptional changes associated with AD progression.

## Introduction

Alzheimer’s disease (AD) is the most prevalent neurodegenerative disease worldwide, leading to progressive cognitive decline, memory loss, and dementia (Alzheimer’s Association, 2020; Wong, 2020; Scheltens et al., 2021). Several environmental and genetic risk factors associated with the development of AD have been recognized (Gatz et al., 2006). Historically, alcohol has received limited attention as a potential risk factor for AD and dementia in general (Gorelick, 2004; Livingston et al., 2017). The scientific literature indicates that alcohol use disorder is associated with a higher risk of all types of dementia, especially early-onset dementia (Mukamal et al., 2003; Xu et al., 2017; Schwarzinger et al., 2018; Koch et al., 2019; Rehm et al., 2019; Kim et al., 2020; Visontay et al., 2021). A large-scale retrospective cohort of French patients found a strong association between alcohol use disorder and dementia (Schwarzinger et al., 2018). Excessive alcohol drinking was particularly associated with early-onset dementia (<65 years of age; Schwarzinger et al., 2018). However, the association between alcohol and AD was not directly addressed (Schwarzinger et al., 2018). In some studies, a history of heavy drinking was associated with an earlier onset of AD and faster cognitive decline (Harwood et al., 2010; Heymann et al., 2016; Chosy et al., 2022).

Here, we explored effects of the interaction of a history of alcohol intoxication with genetic AD susceptibility on cognitive performance and gene expression at the single-cell level. We used triple-transgenic AD (3xTg-AD) mice, which harbor three familial AD (FAD) mutations, including transgenes of the human amyloid precursor protein (APP) Swedish double mutation (KM670/671NL) and the human tau_P301L_ mutation, and knockin of the human presenilin (PS1) M146V mutation (Oddo et al., 2003). We show that repeated alcohol intoxication by exposure to alcohol vapor hastens the onset of cognitive impairment in 3xTg-AD mice. These results were confirmed in independent cohorts of 3xTg-AD mice and 5xFAD mutant mice that harbor three APP and two PS1 familial AD mutations (Oakley et al., 2006). We performed single-nucleus gene expression analyses of the prefrontal cortex (PFC) of 3xTg-AD and wild-type (WT) mice with and without histories of repeated alcohol intoxication. The PFC is affected in AD (Sampath et al., 2017) and in rats with histories of alcohol dependence (Repunte-Canonigo et al., 2007; Trantham-Davidson et al., 2014). In humans with alcohol use disorder, PFC deficits are associated with cognitive impairment and vulnerability to relapse (Sullivan et al., 2000; Wölwer et al., 2001; Tedstone and Coyle, 2004; Rando et al., 2011; Trantham-Davidson et al., 2014).

We observed a broad dysregulation of gene expression that involves neuronal excitability, neurodegeneration, and inflammation, including interferon (IFN) genes. We investigated the differential expression of genes that were previously associated with AD in humans by genome-wide association studies (GWASs) and found that several of them are significantly differentially regulated by alcohol in specific cell populations of both superficial and deep cortical layers of 3xTg-AD mice, suggesting that alcohol intoxication may functionally affect AD GWAS genes in a cell type-specific manner. By comparing gene expression signatures of 3xTg-AD mice with and without histories of repeated alcohol intoxication to gene expression signatures of 3xTg-AD and 5xFAD mice of different ages, we observed that expression patterns of AD mice with a history of alcohol intoxication were more similar to gene expression signatures of older 3xTg-AD and 5xFAD mice with advanced disease and cognitive impairment than were the expression patterns of AD mice without a history of alcohol exposure, suggesting that alcohol promotes transcriptional changes consistent with AD progression.

**Figure 1.**
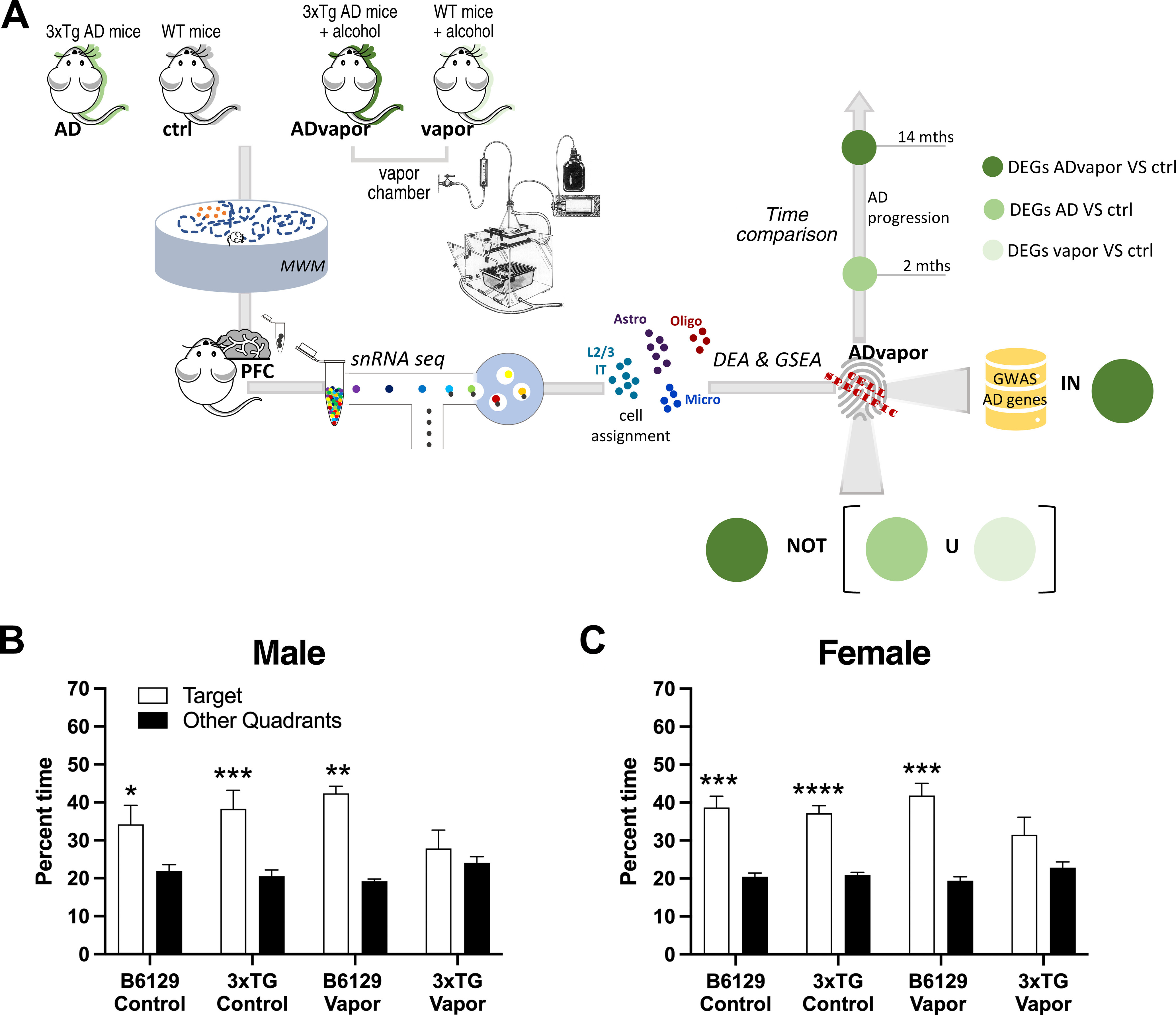
Repeated alcohol intoxication by alcohol vapor exposure hastens the onset of cognitive impairment in 3xTg-AD mice in the Morris water maze (MWM). ***A***, Visual representation of the overall experimental setup. Presymptomatic 3xTg-AD (Tg [APPSwe, tauP301L]1Lfa Psen1tm1Mpm/Mmjax) and WT mice were exposed to repeated cycles of alcohol intoxication by exposure to alcohol vapor with the CIE paradigm. Blood alcohol levels (BALs) are shown in Extended Data Figure 1-2. Mice were then tested in the MWM, and their prefrontal cortices were then processed for snRNA-Seq. ***B***, Male 3xTg-AD mice after exposure to five cycles of chronic intermittent alcohol vapor (3xTG Vapor) showed impaired spatial memory in the MWM, in which they spent comparable time in the target quadrant where the hidden platform was located as in the other quadrants of the pool. Conversely, vapor-exposed WT male C57BL/6;129 (B6129 Vapor), unexposed WT C57BL/6;129 male control (B6129 Control), and 3xTg-AD control (3xTG Control) mice all spent more time in the target quadrant than in the other quadrants. Three-way ANOVA revealed a significant main effect of quadrants (*F*_(1,29)_ = 19.97, *p* < 0.0001) but no main effects of genotype [*F*_(1,29)_ = 1.193, not significant (n.s.)] and treatment (*F*_(1,29)_ = 0.05931, n.s.), and no interactions (quadrant x treatment, *F*_(1,29)_ = 0.05931, n.s.; quadrant x genotype, *F*_(1,29)_ = 1.193, n.s.; treatment x genotype, *F*_(1,29)_ = 3.797, n.s.; quadrant x treatment x genotype, *F*_(1,29)_ = 3.797, n.s.). The data represent the mean ± SEM (*n *=* *5–10). *p <* *0.05, **p <* *0.01, ***p <* *0.001, Fisher’s least significant difference test. ***C***, Female 3xTg-AD mice showed impaired spatial memory in the MWM after six cycles of chronic intermittent alcohol vapor. Three-way ANOVA revealed a significant main effect of quadrants (*F*_(1,35)_ = 54.32, *p* < 0.0001), but no main effects of genotype (*F*_(1,35)_ = 3.139, n.s.) or treatment (*F*_(1,35)_ = 0.1469, n.s.) and no interactions (quadrant x treatment, *F*_(1,35)_ = 0.1471, n.s.; quadrant x genotype, *F*_(1,35)_ = 3.136, n.s.; treatment x genotype, *F*_(1,35)_ = 1.736, n.s.; quadrant x treatment x genotype, *F*_(1,35)_ = 1.734, n.s.). Vapor blood alcohol levels averaged 200 mg/dl and did not differ between genotypes or sexes. The data represent the mean ± SEM (*n *=* *9–10). ***p <* *0.001, ****p <* *0.0001, Fisher’s least significant difference test. See Extended Data Figure 1-1 for MWM results in independent cohorts of male 3xTg-AD mice and 5xFAD mutant mice.

This study aimed to explore transcriptional correlates of the interaction of excessive alcohol intake and genetic AD susceptibility at the single-cell level in a murine model of AD. We found that a history of repeated alcohol intoxication promotes the emergence of spatial learning and memory impairments in presymptomatic 3xTg-AD mice that harbor genetic mutations underlying familial AD and induces PFC transcriptional changes associated with AD progression (Fig. 1*A*). We provide a single-nucleus RNA-sequencing (snRNA-Seq) dataset of the PFC of 3xTg-AD and WT mice with and without a history of repeated alcohol intoxication as a resource to explore gene expression and gene network dysregulations that underlie the detrimental role of alcohol in AD progression at the single-cell level.

**Figure 2.**
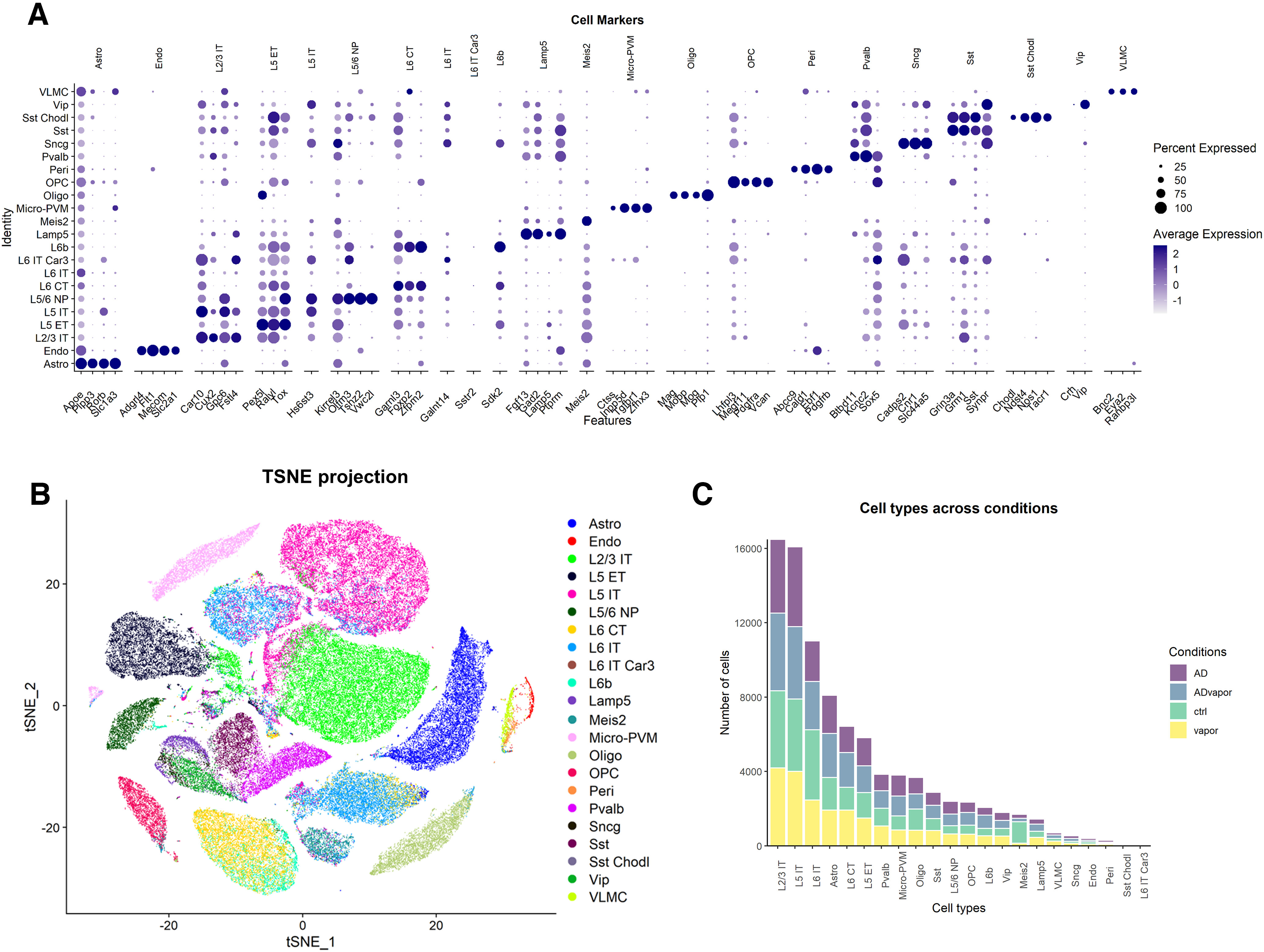
Cytological summary of snRNA-Seq of 3xTg-AD and WT mice with and without a history of repeated alcohol intoxication recapitulates cell type-specific marker genes. ***A***, Dot plot of the expression and presence of selected known gene markers for specific cell types (columns) in cells defined by Azimuth reference-based cell identity assignments (rows). ***B***, t-SNE projection of the entire dataset, showing the identified cell identities. ***C***, Stacked bar plot of the number of cells in each cell type, distributed according to the four experimental conditions (Extended Data Fig. 2-1, list of abbreviations of cell types, Fig. 2-2, distribution of cell types in the four experimental conditions).

10.1523/ENEURO.0456-22.2023.f1-1 Figure 1-1 Repeated alcohol intoxication by alcohol vapor exposure hastens the onset of cognitive impairment in 3xTg-AD and 5xFAD mice in the Morris water maze (MWM). (A) A separate cohort of male 3xTg-AD mice showed impaired spatial memory after five cycles of chronic intermittent alcohol vapor in the MWM, in which they spent comparable time in the target quadrant where the hidden platform was located as in the other quadrants of the pool (3xTG Vapor). Conversely, 3xTg-AD mice not exposed to alcohol (3xTG Control) and WT mice either exposed or not exposed to alcohol vapor spent significantly more time in the target quadrant compared to the other quadrants (B6129 Control and B6129 Vapor). Three-way analysis of variance revealed a significant main effect of quadrants (*F*_1,52_ = 11.64, *p* < 0.01) and a quadrant genotype interaction (*F*_1,52_ = 4.201, p < 0.05). The data represent the mean ± SEM. *n* = 7-20. *p < 0.05, **p < 0.01, Fisher's Least Significant Difference test. (B) Similarly, male 5xFAD mice also showed impaired spatial memory after three cycles of chronic intermittent alcohol vapor in the MWM (5xFAD Vapor), whereas 5xFAD mice not exposed to alcohol (5xFAD Control) and WT mice either exposed or not exposed to alcohol vapor (C57Bl6/J Control and C57Bl6/J Vapor) spent significantly more time in the target quadrant compared to the other quadrants. Three-way analysis of variance revealed a significant main effect of genotype (*F*_1,52_ = 28.75, *p*< 0.0001). The data represent the mean ± SEM. *n* = 10-20. *p < 0.05, **p < 0.01, ***p < 0.001, Fisher's Least Significant Difference test. BALs are shown in Fig 1-2. Download Figure 1-1, TIFF file.

10.1523/ENEURO.0456-22.2023.f1-2 Extended Data Figure 1-2 Blood alcohol levels (mg/dl) of alcohol vapor-exposed mice in the study. Download Figure 1-2, XLSX file.

## Materials and Methods

### Mice

3xTg-AD mice on a B6;129 genetic background (https://www.jax.org/strain/004807) and 5xFAD mice on the C57BL/6J genetic background (https://www.jax.org/strain/008730) were obtained from The Jackson Laboratory.

### Alcohol intoxication

Alcohol vapor was created by dripping 95% alcohol into a 2000 ml Erlenmeyer vacuum on a warming tray (50°C). Alcohol vapor was independently introduced into each sealed chamber through a stainless steel manifold (11 L/min). The chronic intermittent ethanol (CIE) vapor group was injected with 1.75 g/kg ethanol plus 68.1 mg/kg pyrazole (alcohol dehydrogenase inhibitor) and placed in the chambers to receive intermittent vapor for 4 d (vapor on, 16 h; vapor off, 8 h). Following the third 16 h bout of vapor, the mice were removed, and tail blood was sampled for blood alcohol level (BAL) determination. Target blood alcohol levels were 150–200 mg/dl, measured on a gas chromatography system (model 7820A, Agilent) coupled to a headspace flame ionization sampler system (model 7697A, Agilent). BALs are shown in Extended Data Figure 1-2. Following day 4 of exposure, the mice were allowed 72 h of undisturbed time. The mice were then re-exposed to alcohol vapor for another week. The mice received 1 week off, and then this cycle was repeated for a total of 5–6 weeks of CIE for the male and female 3xTg-AD/B6129 mice, 5 weeks of CIE for the replicate male-only 3xTg-AD/B6129 mice, and 3 weeks of CIE for the male 5xFAD/C57BL6J mice. The control groups were injected with 68.1 mg/kg pyrazole in saline and placed in chambers delivering air for the same periods as the CIE groups. 3xTg-AD/B6129 mice and their respective control mice were euthanized at 22–24 weeks of age. 5xFAD/C57BL6J mice and their control mice were euthanized at 22 weeks of age.

### Morris water maze test

The Morris water maze test was used to assess spatial learning and memory in rodents (Morris, 1984; Puzzo et al., 2014). The mice were placed in a circular tub that was filled with opaque water (21 ± 1°C), and they learned over repeated trials to locate a hidden platform onto which they could sit and escape from swimming. Each animal underwent two trials per day for 4 d, with a fixed platform location but a random start position. After being placed in the water, each animal was allowed to swim until it found the platform or 90 s elapsed, at which point the experimenter gently guided the mouse to the platform. A probe trial was given after the completion of training (day 5), in which the platform was removed from the water maze and the animal was allowed to swim freely for 90 s. Following each trial, the mice were placed in a clean cage lined with a warm towel for 10 min. The same protocol was used in each separate experiment, and the first day of acquisition was always 72 h following removal from the vapor chambers (mice removed from vapor on Friday, and testing was initiated on the following Monday). The same highly trained technician ran each of the studies, starting 1 h into the dark phase of the light/dark cycle. Data were analyzed using Ethovision software (Noldus).

### snRNA-Seq and computational methods

Male 3xTg-AD mice were euthanized under deep isoflurane anesthesia, and freshly dissected brain regions were snap frozen. The medial PFC, including the prelimbic and infralimbic cortices, from ∼1 mm coronal sections (bregma, 2.7–1.8 mm), were processed for snRNA-Seq by Singulomics using the 10x gene expression platform (Chromium, Genomics).

Raw 10x Genomics reads from 16 samples (*n *=* *4 3xTg-AD and WT mice with and without histories of repeated alcohol intoxication) were aligned on the *Mus musculus* genome version mm10 (downloaded August 13, 2021) using Cell Ranger 6.1.1 based on the STAR aligner (Dobin et al., 2013). The sparse H5 matrix object generated by Cell Ranger and containing cell-by-cell and gene-by-gene estimated read counts was loaded into R using the Seurat 4.1.1 package (Shalek et al., 2014). Cell assignment was performed using the Azimuth pipeline (Hao et al., 2021) with default parameters using the reference dataset from the work of Yao et al. (2021). Dimensionality reduction of the dataset was performed using the t-SNE (t-distributed stochastic neighbor embedding) algorithm as implemented by the R package Rtsne. To perform differential expression analysis, we used the R package DESeq2 (Love et al., 2014) after coalescing all gene counts belonging to the same cell type/sample combination.

The most significantly differentially expressed genes were selected among the differentially expressed coding genes (obtained by removing genes that did not have a canonical name yet; i.e., gene names starting with “Gm-” and RIKEN genes) for each contrast, filtering by adjusted *p* value (*p *≤ 0.05) and by DESeq2 baseMean value [log10(b) > 1] to exclude low expression genes. The *pheatmap* function of the *ComplexHeatmap* R package (version 2.12.1; Gu et al., 2016) was used to plot up to the top five genes for each cell type. The selected genes were color coded according to the log2 fold change values if their log10 baseMean was higher than unit; otherwise, they were colored in gray.

Gene Set Enrichment Analysis (GSEA; Subramanian et al., 2005) was performed using the *fgsea* function on the following sections of the Broad Institute MsigDB collection: KEGG, WikiPathways, Gene Ontology [BP (biological process), CC (cellular component), and MF (molecular function)], and Hallmark Pathways. To render the enriched pathways with heatmaps, nearly identical pathways were collapsed using the *fgsea* function *collapsePathways* with the default parameters on the GSEA results filtered for adjusted *p *<* *0.01 (*fgsea* version 1.22.0). The top five main pathways (not reducible to each other) for each cell type were selected. Wherever the same pathway was present in multiple databases (e.g., Ribosome), the corresponding database was also indicated (e.g., KEGG Ribosome and GO:CC Ribosome).

All other statistical operations and data analysis steps were performed using R version 4.2.1 (Giorgi et al., 2022), unless otherwise specified.

### Cell type-specific analysis of AD-associated GWAS genes

Alzheimer’s disease-associated genes were retrieved from the NHGRI-EBI (National Human Genome Research Institute-European Bioinformatics Institute) catalog (Buniello et al., 2019; accessed September 29, 2022) to download the following four traits: “Alzheimer Disease” (MONDO_0004975), “Alzheimer’s Disease Biomarker Measurements” (EFO-0006514), “Late-Onset Alzheimer’s Disease” (EFO-1001870), and “Alzheimer’s Disease Neuropathologic Change” (EFO-0006801). Associations with *p *≤* *9 × 10^−6^ were considered significant, and the genes reported to be associated by the authors of each GWAS study were used as gene lists after removing duplicate genes as well as GWAS *loci* in intergenic regions. Human genes were converted to mouse orthologs using the biomaRt R package (Durinck et al., 2009). This list was overlapped with the differentially expressed genes (adjusted *p *≤* *0.05) in each cell type for the contrast 3xTg-AD mice with a history of repeated alcohol intoxication by alcohol vapor exposure versus WT control mice not exposed to alcohol.

### Comparison with Yin et al. (2020)

The transcriptional profiling of the 3xTg-AD mouse model from 2 to 14 months of age was previously reported by Yin et al. (2020). Differentially expressed genes between 3xTg-AD and WT mouse insular cortex samples for the earliest and latest time points separately were obtained from Yin et al. (2020, refer to their Supplementary Table S1). GSEA was performed using the *corto* version 1.1.11 package (Mercatelli et al., 2020) considering the upregulated genes (log2 fold change > 0) at 2 and 14 months as gene sets to determine their enrichment in our pseudo-bulk signatures of 3xTg-AD mice with histories of repeated alcohol intoxication versus WT controls and of 3xTg-AD mice without alcohol exposure versus WT mice without alcohol exposure.

### Comparison with Forner et al. (2021)

Forner et al. (2021) performed a phenotypic characterization of the 5xFAD mouse model including bulk RNA-Seq experiments at different time points in 5xFAD and BL6 strains. The data were deposited in Gene Expression Omnibus (GEO; accession no. GSE168137). The count matrix was downloaded, and differential expression analyses were performed with the DESeq2 package between cortex samples from AD and BL6 strains at 4 and 18 months of age. Then GSEA was performed using *corto* to determine whether the gene set composed of the upregulated genes (adjusted *p *≤* *0.05; log2 fold change ≥ 1) at the two time points separately were enriched for the pseudo-bulk signatures of our 3xTg-AD mice with a history of repeated alcohol intoxication by alcohol vapor exposure versus WT control mice not exposed to alcohol and the 3xTg-AD mice without alcohol exposure versus WT mice without alcohol exposure.

### Data availability

snRNA-Seq data of the PFC of 16 male 3xTg-AD and WT mice with and without histories of repeated alcohol intoxication by exposure to alcohol vapor were deposited in the National Center for Biotechnology Information Gene Expression Omnibus and are accessible through GEO Series accession no. GSE218309 (https://www.ncbi.nlm.nih.gov/geo/query/acc.cgi?acc=GSE218309).

## Results

### Repeated alcohol intoxication by exposure to alcohol vapor hastens the emergence of cognitive impairment in 3xTg-AD mice

3xTg-AD mice develop progressive cognitive impairments in spatial memory (Oddo et al., 2003; Billings et al., 2005). To test the hypothesis that a history of repeated alcohol intoxication hastens cognitive impairment progression in the setting of genetic AD susceptibility, we exposed presymptomatic 3xTg-AD and WT mice (age, 2 months) to repeated cycles of alcohol intoxication by exposure to alcohol vapor with the established CIE paradigm (Becker and Hale, 1993; Becker and Lopez, 2004). We then examined spatial memory in the Morris water maze memory test in 3xTg-AD and WT mice during protracted withdrawal after exposure to repeated cycles of alcohol intoxication (5–6 weeks of total exposure with 2 weeks on, 1 week off, repeated three times) and unexposed control mice of both genotypes when they were 4.25 (males) and 4.5 (females) months of age. The results showed that repeated cycles of alcohol intoxication with the CIE paradigm hastened the onset of cognitive impairment in 3xTg-AD mice (Fig. 1*B,C*). 3xTg-AD mice not exposed to alcohol vapor and WT mice both exposed and not exposed to alcohol vapor were not impaired and performed similarly in the Morris water maze (Fig. 1*B,C*). These results were confirmed in independent cohorts of male 3xTg-AD mice and 5xFAD mutant mice, which harbor three APP and two PS1 FAD mutations (Oakley et al., 2006; Extended Data Fig. 1-1). Body weights increased across the experiment in all groups by 1.5 ± 0.2 g, with no differences among groups. These results lend support to the hypothesis that a history of excessive alcohol intake promotes AD progression in the setting of genetic susceptibility and show that repeated alcohol intoxication by exposure to alcohol vapor is a robust method to model the interaction of excessive alcohol intake with AD susceptibility.

### Ultra-high-definition molecular survey of the interaction of alcohol with Alzheimer’s disease: snRNA-Seq of 3xTg-AD and WT mice with and without a history of repeated alcohol intoxication

To investigate the gene signature and disease-related transcriptional changes induced by repeated cycles of alcohol intoxication sufficient to promote cognitive impairment in 3xTg-AD mice, we performed snRNA-Seq of the PFC of 3xTg-AD and WT mice exposed to alcohol vapor and unexposed mice of both genotypes at 4.75–5 months of age (4–7 d following behavioral testing; 11–14 d following the final alcohol vapor exposure) on the 10x Genomics platform. We sampled 516,560 cells. After quality control filtering (see Materials and Methods), we obtained 113,242 cells with a mean of 1986.7 detected genes per cell. The Azimuth reference-based cell identity assignment was used for cell type identification, confidently assigning 91,898 cells to 22 cytological types according to established mouse neocortex molecular taxonomy (Yao et al., 2021; Fig. 2*A*, Extended Data Fig. 2-1). Gene markers for the majority of cell types allowed us to clearly and robustly discern the underlying histology, in particular for non-neuronal cell types, including endothelial cells, oligodendrocytes, and vascular leptomeningeal cells, among others (Fig. 2*A*,*B*).

10.1523/ENEURO.0456-22.2023.f2-1 Figure 2-1 Abbreviations for cell types. Download Figure 2-1, DOCX file.

10.1523/ENEURO.0456-22.2023.f2-2 Figure 2-2 Stacked bar plot showing the distribution of cell types in the four experimental conditions. Total number of cells in the dataset are shown at the top of each bar. Download Figure 2-2, TIF file.

The visualization of single-nucleus transcriptomes in t-SNE showed that nuclei of the 22 detected cell types were loosely distributed in transcriptionally separated clusters (Fig. 2*B*). Overall, the main source of variance in our dataset was the cell type, reflected by the cell marker landscape (Fig. 2*A*). Cells assigned to astrocyte, oligodendrocyte, oligodendrocyte progenitor cell (OPC), and microglia lineages were clearly distinct in well defined clusters, whereas GABAergic neurons (Pvalb, Sst, Sst Chodl, Sncg, Vip, and Lamp5) constitute a separate cell population. The most abundant cell populations were glutamatergic neurons, intratelencephalon projecting, from layers 2, 3, 5, and 6 (Fig. 2*B*,*C*).

The distribution of cell types in the four experimental conditions was generally well balanced (Fig. 2*C*, Extended Data Fig. 2-2). Overall, we found that approximately two-thirds of differentially expressed transcripts were represented by protein-coding genes, and approximately one-third by noncoding genes (Extended Data Fig. 3-1).

### Cell type-specific genes associated with a history of alcohol intoxication in the PFC of 3xTg-AD mice highlight candidate mechanisms of AD disease progression

**Figure 3.**
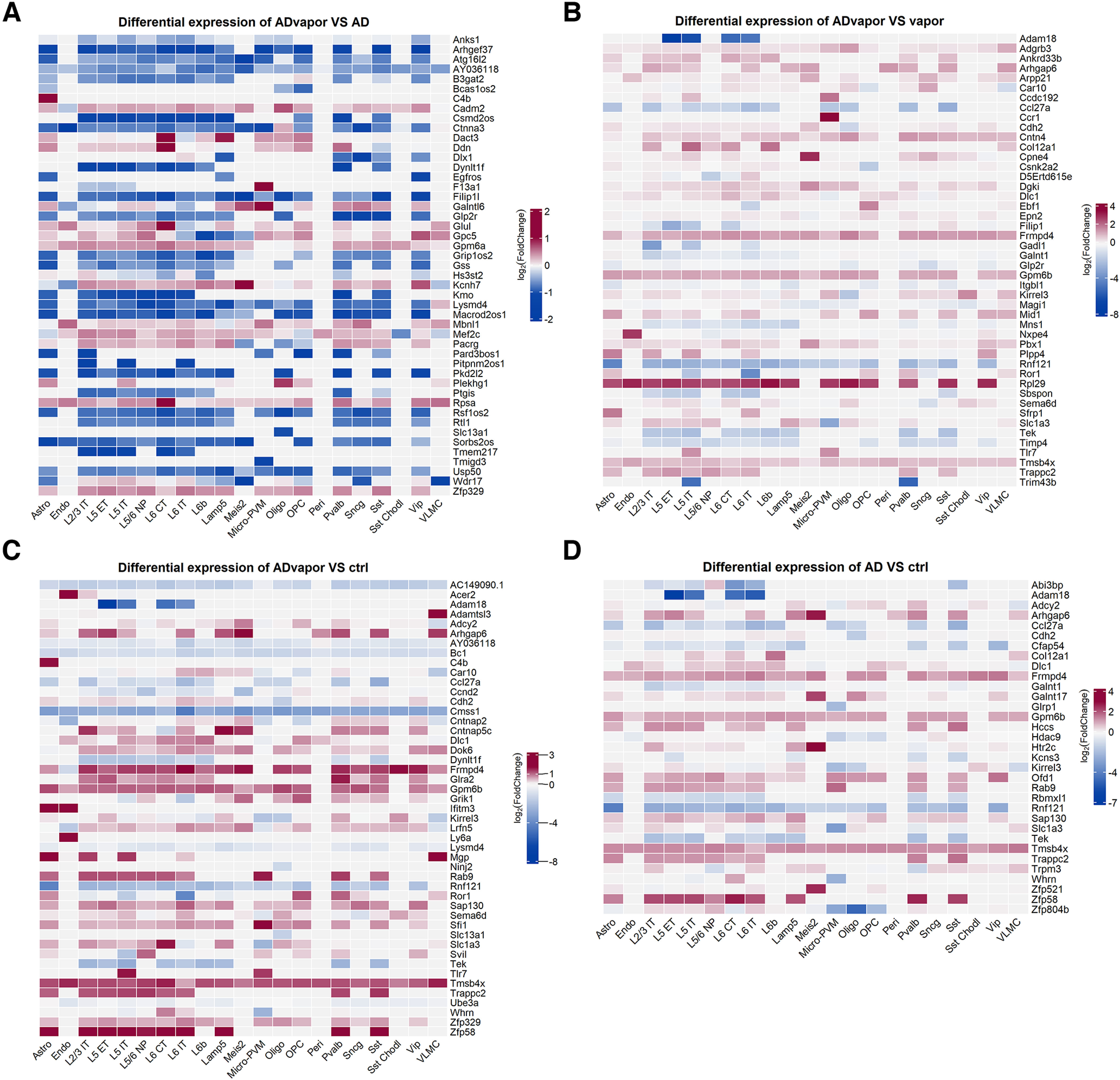
Cell type-specific gene expression changes in 3xTg-AD and WT mice with and without a history of repeated alcohol intoxication. ***A–D***, Heatmaps of the most significantly differentially expressed coding genes in the following contrasts: 3xTg-AD mice with a history of repeated alcohol intoxication by exposure to alcohol vapor (ADvapor) versus 3xTg-AD control mice not exposed to alcohol (AD; ***A***); 3xTg-AD mice with a history of repeated alcohol intoxication (ADvapor) versus WT mice with a history of alcohol intoxication (vapor; ***B***); 3xTg-AD mice with a history of repeated alcohol intoxication (ADvapor) versus WT mice not exposed to alcohol (ctrl; ***C***); 3xTg-AD mice without alcohol exposure (AD) versus WT mice not exposed to alcohol (ctrl; ***D***; Extended Data Fig. 3-1, distribution of differentially expressed genes in each cell type in the four contrasts).

**Figure 4.**
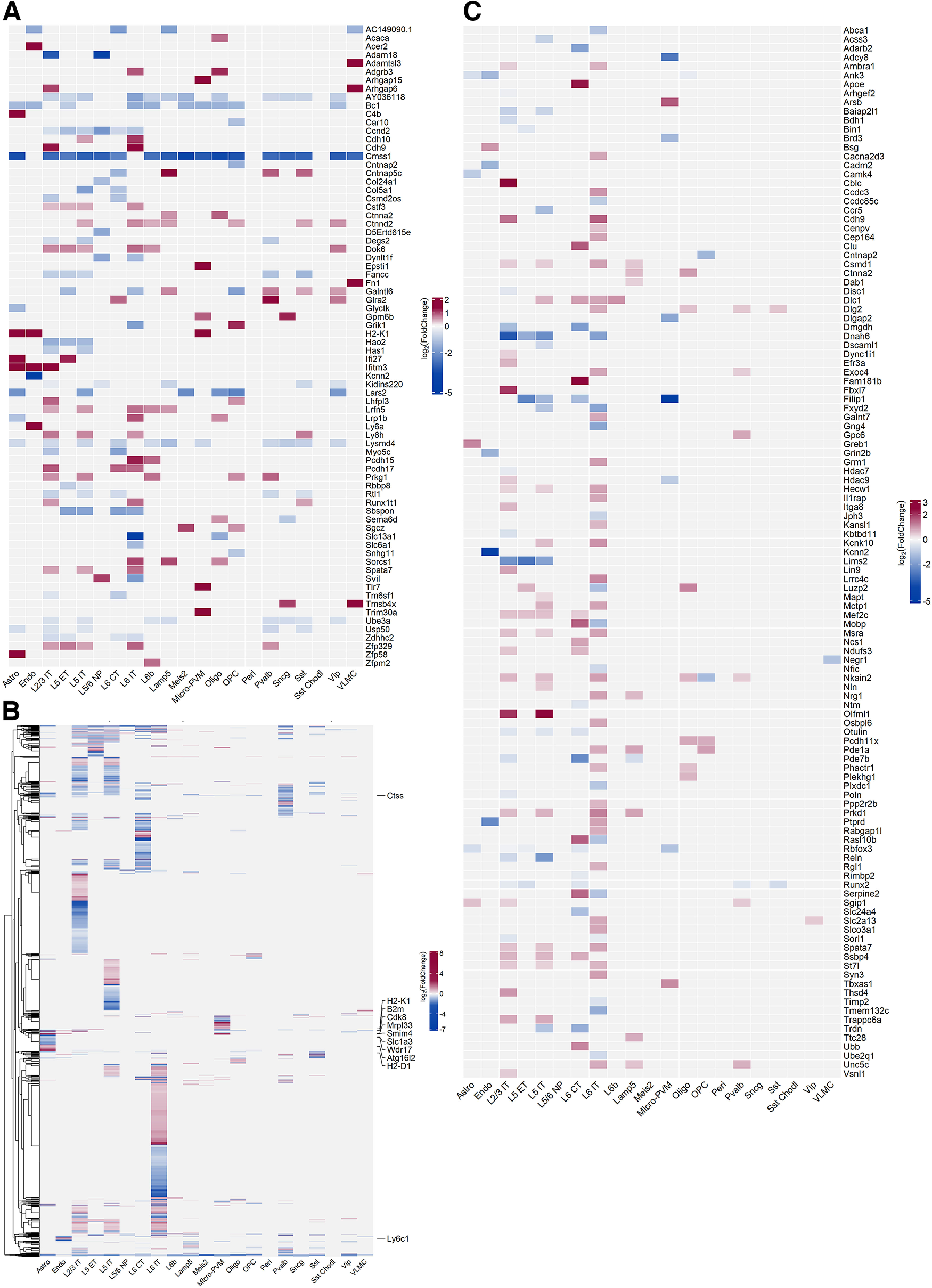
Selected and global transcriptomics landscape of cell type-specific differential gene expression in 3xTg-AD mice with a history of repeated alcohol intoxication. ***A***, Heatmap of the top five most differentially expressed coding genes per cell type in 3xTg-AD mice with a history of repeated alcohol intoxication by alcohol vapor exposure versus WT control mice not exposed to alcohol but not significantly differentially expressed in 3xTg-AD mice without histories of alcohol intoxication or WT mice with a history of alcohol intoxication versus WT control mice not exposed to alcohol. ***B***, Heatmap of all significantly differentially expressed genes per cell type in 3xTg-AD mice with a history of repeated alcohol intoxication by alcohol vapor exposure versus WT control mice not exposed to alcohol but not significantly differentially expressed in 3xTg-AD mice without histories of alcohol intoxication or WT mice with a history of alcohol intoxication versus WT control mice not exposed to alcohol. The list is available in Extended Data Figure 4-1. Selected genes are labeled. ***C***, Heatmap of differential expression of genes associated with AD from GWASs (see Materials and Methods) in the contrast of 3xTg-AD mice with a history of repeated alcohol intoxication by alcohol vapor exposure versus WT control mice not exposed to alcohol (Extended Data Fig. 4-1, tabular results reporting significantly differentially expressed genes related to panels ***A*** and ***B***).

Differentially expressed genes are shown in Figure 3. Cmss1 (Cms1 Ribosomal Small Subunit Homolog) was selectively downregulated in 3xTg-AD mice exposed to alcohol versus WT mice without alcohol exposure but not in 3xTg-AD mice and WT mice exposed to alcohol versus WT controls in multiple neuronal and non-neuronal cell types, including L5/6 nonpeptidergic (NP), L5 external tufted (ET), L2/3 IT, L5 IT, L6b, L6 corticothalamic (CT), VIP, parvalbumin, Meis2, Sncg and SSt interneurons, Lamp5, astrocytes, microglia-perivascular macrophages (PVMs), oligodendrocytes, and OPCs (Fig. 4*A*, Extended Data Fig. 4-1). Several ribosomal proteins were selectively decreased in 3xTg-AD mice exposed to alcohol versus WT controls but not in 3xTg-AD mice and WT mice exposed to alcohol versus WT controls in L6 IT neurons, including Rpl23a, Rpl13a, Rpl35a, Rpl36a, Rpl37, and Mrpl33 [Mrpl33 also decreased in Sst and L2/3 intratelencephalic (IT) neurons]. Conversely, ribosomal proteins were generally unchanged or increased in other cell types (Fig. 4*B*, Extended Data Fig. 4-1). The mitochondrial leucyl-tRNA synthetase (Lars2), implicated in mitochondrial protein synthesis, lactate utilization (Capriglia et al., 2021), episodic memory in aging (Ramanan et al., 2015), bipolar disorder, and schizophrenia (Munakata et al., 2005), was selectively downregulated in multiple neuronal and non-neuronal cell types, including L5 IT, Meis2, VIP, astrocytes, oligodendrocytes, and OPC, in 3xTg-AD mice exposed to alcohol versus WT controls but not in 3xTg-AD mice and WT mice exposed to alcohol versus WT controls (Fig. 4*A*, Extended Data Fig. 4-1).

10.1523/ENEURO.0456-22.2023.f3-1 Figure 3-1 Distribution of differentially expressed genes in each cell type in the four contrasts. ***A***, Differential expression analysis in four contrasts, representing the total number of genes differentially expressed at an adjusted *p *≤* *0.05, divided into upregulated (up) and downregulated (dn). ***B***, As in ***A***, with numbers referring to protein-coding genes only. ***C***, As in ***A***, with numbers referring to noncoding genes. AD, 3xTg-AD mice; Advapor, 3xTg-AD mice with a history of repeated alcohol intoxication by alcohol vapor exposure; ctrl, WT control mice not exposed to alcohol; vapor, WT control mice with a history of repeated alcohol intoxication by alcohol vapor exposure. Download Figure 3-1, TIF file.

10.1523/ENEURO.0456-22.2023.f4-1 Figure 4-1 Table of genes significantly (adjusted *p *≤* *0.05) differentially expressed in specific cell types in the contrast of 3xTg-AD mice with a history of repeated alcohol intoxication by exposure to alcohol vapor (ADvapor) versus WT mice not exposed to alcohol (ctrl) and not significantly in 3xTg-AD mice without alcohol exposure (AD) and WT mice with a history of alcohol intoxication (vapor) versus ctrl, according to DESeq2 analysis and subsequent filtering. Download Figure 4-1, XLS file.

A broad increase in the transcription of cadherins was seen in excitatory neurons of both deep and superficial layers of 3xTg-AD mice exposed to alcohol versus WT controls but not in 3xTg-AD mice and WT mice exposed to alcohol versus WT controls (Fig. 4, Extended Data Fig. 4-1). Particularly, L6 IT neurons showed increases in the transcription of cadherins, including Pcdh15, Pcdh17, Pcdh9, Cdh8, Cdh9, Cdh10, Cdh11, and Cdh13, whereas Pcdh17 and Pcdh19 were upregulated in L6 CT neurons. L5 IT neurons showed increased Cdh10. L2/3 IT neurons showed the increased transcription of Cdh9 and Pcdh17, and L6b neurons showed an increase in Pcdh15. Cdh2 (N-cadherin) was significantly decreased in oligodendrocytes of 3xTg-AD mice exposed to alcohol versus WT controls (adjusted *p *=* *6.87e-09; log_2_ fold change = −1.70; Fig. 3*C*) as well as of 3xTg-AD mice not exposed to alcohol versus WT controls (adjusted *p *=* *3.35e-07; log_2_ fold change = −1.60; Fig. 3*D*) and of 3xTg-AD mice exposed to alcohol versus WT mice exposed to alcohol (adjusted *p *=* *4.54e-05; log2 fold change = −1.40; Fig. 3*B*). Cdh2 showed upregulation in other cell types (i.e., L6 IT; Fig. 3).

Parvalbumin and VIP interneurons and L6 CT neurons showed the increased transcription of Glra2 (glycine receptor α2; Fig. 4*A*, Extended Data Fig. 4-1), and VIP interneurons showed the increased expression of Grid2 (glutamate receptor GluRδ2; Extended Data Fig. 4-1) in 3xTg-AD mice exposed to alcohol versus WT controls but not in 3xTg-AD mice versus WT controls or in WT mice exposed to alcohol versus WT controls.

Contactin-associated protein 5c (Cntnap5c), a member of a cell adhesion protein family implicated in interneuron development and function (Bridi et al., 2017), was selectively upregulated in 3xTg-AD mice exposed to alcohol versus WT controls but not in 3xTg-AD mice or WT mice exposed to alcohol versus WT controls in parvalbumin, Lamp5, and Sst neurons, and decreased in L6 CT neurons (Fig. 4*A*). Its paralog Cntnap5b increased in VIP GABAergic neurons in 3xTg-AD mice exposed to alcohol versus WT controls but not in 3xTg-AD mice or WT mice exposed to alcohol versus WT controls (Extended Data Fig. 4-1). Another member of the protein family, Cntnap2, was decreased in OPC (Fig. 4*A*, Extended Data Fig. 4-1).

α-Synuclein (Snca) was slightly but significantly increased in 3xTg-AD mice exposed to alcohol versus WT controls but not in 3xTg-AD mice or WT mice exposed to alcohol versus WT controls of pyramidal neurons in L5 and L2/3 (L2/3 IT, L5 IT; Extended Data Fig. 4-1).

Slc1a3, encoding for the excitatory amino acid transporter 1 (EAAT1), was among the genes significantly increased by repeated alcohol intoxication in astrocytes of 3xTg-AD mice exposed to alcohol versus WT controls but not in 3xTg-AD mice or WT mice exposed to alcohol versus WT controls (Extended Data Fig. 4-1). In microglia, Slc1a3 was significantly downregulated in both 3xTg-AD mice exposed to alcohol and 3xTg-AD mice not exposed to alcohol versus WT control mice not exposed to alcohol (Fig. 3*C*,*D*, respectively).

In 3xTg-AD mice exposed to alcohol versus WT controls but not in 3xTg-AD mice or WT mice exposed to alcohol versus WT controls, complement C4b and metallothionein-1 (Mt1) were selectively increased in astrocytes, whereas among IFN-regulated genes, Ifitm3 was upregulated in astrocytes, endothelial cells, and L2/3 IT, and Ifi27 was upregulated in astrocytes and L5 ET (Fig. 4*A*, Extended Data Fig. 4-1).

Genes with decreased expression in astrocytes in 3xTg-AD mice exposed to alcohol versus WT controls but not in 3xTg-AD mice or WT mice exposed to alcohol versus WT controls included the following: the WD repeat-containing protein Wdr17, abundantly expressed in astrocytes and associated with macular degeneration (Stöhr et al., 2002); Smim4, which may be involved in mitochondrial toxicity (Dennerlein et al., 2021); and autophagy-related 16 like 2 (Atg16l2), a proposed peripheral marker of AD (Qin et al., 2022) that has been shown to be a negative regulator of the NOD (nucleotide-binding oligomerization domain)-like receptor family, pyrin domain containing 3 (NLRP3) inflammasome (Wang et al., 2022) and thus may contribute to neuroinflammation (Fig. 4*B*, Extended Data Fig. 4-1).

Among the most increased genes in microglia-PVM in 3xTg-AD mice exposed to alcohol versus WT controls but not in 3xTg-AD mice versus WT controls and WT mice exposed to alcohol versus WT controls are contributors to neuroinflammation in the setting of AD and alcohol abuse, including the Toll-like receptor 7 (Tlr7), which was found to be increased in the brains of humans with alcohol use disorder (Coleman et al., 2017); Epsti1, a regulator of inflammatory gene expression in macrophages (Kim et al., 2018); cathepsin S, a lysosomal protease that is also active outside the lysosome implicated in inflammation (Kim et al., 2017) and AD (Lowry and Klegeris, 2018); H2-K1, H2-D1, and the Rho guanosine triphosphatase-activating protein 15 (Arhgap15), a regulator of the β2-integrin Mac-1 (Margraf et al., 2020); the IFN-induced protein Apobec3; and Gpm6b, which was also upregulated in the parietal cortex in AD brains (Patel et al., 2019; Fig. 4*A*,*B*, Extended Data Fig. 4-1).

The cyclin-dependent kinase Cdk8 was selectively downregulated in 3xTg-AD mice exposed to alcohol versus WT controls but not in 3xTg-AD mice or WT mice exposed to alcohol versus WT controls in multiple non-neuronal cell types, including both astrocytes and microglia-PVM, VLMC (ventral laryngeal motor cortex), and oligodendrocytes, and similar trends were present in some neuronal populations. Cdk8 is part of a protein complex that regulates RNA polymerase II, is involved in regulating multiple cellular states, including development, metabolism, and innate immunity (Dannappel et al., 2018), and was also decreased in the liver by chronic alcohol exposure (Song et al., 2017).

In endothelial cells, several upregulated genes in 3xTg-AD mice exposed to alcohol versus WT controls but not in 3xTg-AD mice or WT mice exposed to alcohol versus WT controls were previously found to be upregulated in aged capillaries, including innate immunity genes, such as Ly6a, Ly6c1, B2m, H2-K1, H2-D1, and Ifitm3 (Chen et al., 2020).

Overall, neurons in both superficial and deep layers of the PFC of 3xTg-AD mice were highly transcriptionally responsive to a history of alcohol intoxication (Fig. 4*B*). Genes previously associated with AD in humans by GWASs were found to be differentially regulated in specific neuronal populations in both deep and superficial layers (Fig. 4*C*), particularly in L2/3 IT, L5 IT, and L6 IT.

### Differentially expressed genes associated with a history of alcohol intoxication in the prefrontal cortex of 3xTg-AD mice indicate that a history of alcohol intoxication promotes AD disease progression

**Figure 5.**
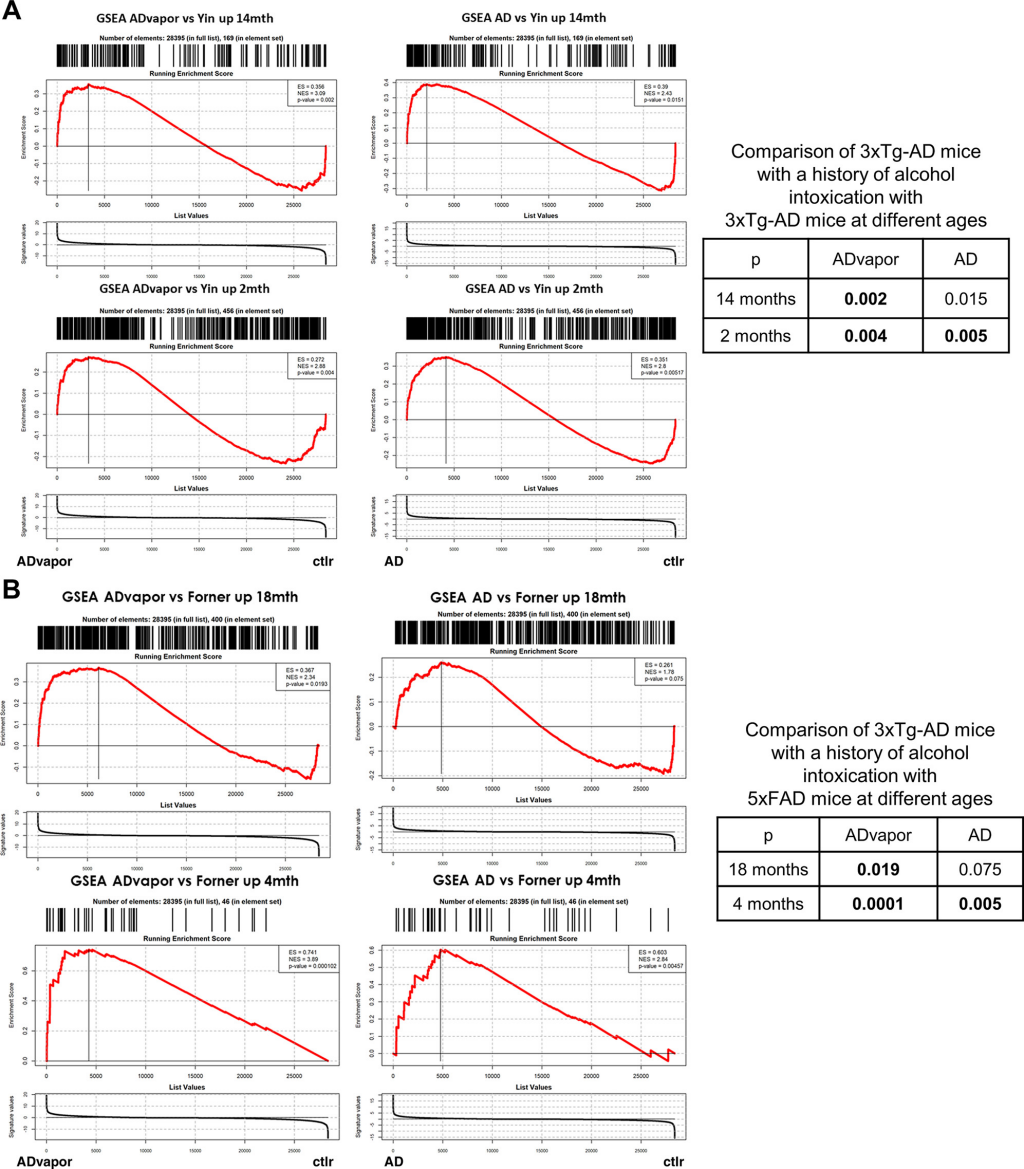
Transcriptional evidence that a history of alcohol intoxication promotes AD progression. ***A***, GSEA analysis was used to compare genes that were significantly upregulated in 3xTg-AD mice (Yin et al., 2020) at 14 months (top) and 2 months (bottom) with the transcriptome-wide signature defined by our contrast of 3xTg-AD mice with a history of repeated alcohol intoxication by alcohol vapor (ADvapor) versus WT control (left) or 3xTg-AD mice not exposed to alcohol versus WT control (right). All cell types were used to define the signature, expressed as -log_10_(*p*)*sign(log_2_FC). The results indicated greater similarity between 3xTg-AD mice at 14 months by Yin et al. (2020) for the ADvapor versus WT control contrast than for the AD versus WT control contrast. ***B***, GSEA analysis was used to compare genes that were significantly upregulated in 5xFAD mice at 18 months (top) and 4 months (bottom) from the study by Forner et al. (2021) with the transcriptome-wide signature defined by our contrast of 3xTg-AD mice with a history of repeated alcohol intoxication by alcohol vapor (ADvapor) versus WT control (left) and 3xTg-AD mice not exposed to alcohol versus WT control (right), like in panel A. The analysis showed positive and significant similarity between the AD model at 18 months for the ADvapor versus WT control contrast but not for the AD versus WT control contrast.

To test the hypothesis that a history of repeated alcohol intoxication promotes disease progression in 3xTg-AD mice, we compared transcriptional responses of our dataset with those generated with age-dependent AD progression data by Yin et al. (2020). We searched for similarities in genes upregulated in a dataset of the insular cortex of 3xTg-AD mice at 14 months of age and 2 months of age in the study by Yin et al. (2020) by overlaying them onto the pseudo-bulk signature in our experiment, defined by the comparison of the PFC of 3xTg-AD mice exposed to alcohol to WT mice without alcohol exposure using the GSEA algorithm (Subramanian et al., 2005; Fig. 5*A*). The results showed positive and significant similarity between the signature of 3xTg-AD mice at 14 months by Yin et al. (2020) and 3xTg-AD mice exposed to alcohol versus WT control mice in the present study but lower similarity for the signature of 3xTg-AD mice not exposed to alcohol versus WT control mice (Fig. 5*A*). We then conducted the same analysis with the signature of genes upregulated in 5xFAD mice at 18 and 4 months of age in a cerebral cortex dataset from the study by Forner et al. (2021; Fig. 5*B*). The GSEA calculated for the signature of 3xTg-AD mice exposed to alcohol versus WT controls in the present study resulted in positive and significant similarity to signatures of 5xFAD mice at 18 and 4 months of age from the study by Forner et al. (2021), whereas 3xTg-AD mice not exposed to alcohol versus WT controls in this work were significantly enriched for genes characterizing the signature of 5xFAD mice at 4 months but not at 18 months. However, the reference set of Forner et al. (2021) at 4 months corresponds to 46 genes compared with 400 elements at 18 months.

These comparisons indicate that the gene expression signature induced by repeated cycles of alcohol intoxication in presymptomatic 3xTg-AD mice in the present study are significantly more similar to 3xTg-AD mice (Yin et al., 2020) and 5xFAD mice (Forner et al., 2021) that are older and have more advanced disease and cognitive impairment than the gene expression signature of 3xTg-AD mice not exposed to alcohol. Therefore, and with the caveats presented by cross-dataset comparison analyses, this analysis suggests that repeated alcohol intoxication promotes transcriptional changes consistent with AD progression.

### Pathway analysis by GSEA in the PFC of 3xTg-AD mice with a history of alcohol intoxication indicates increased inflammation, cell type-specific vulnerability, and activation of compensatory and adaptive mechanisms

**Figure 6.**
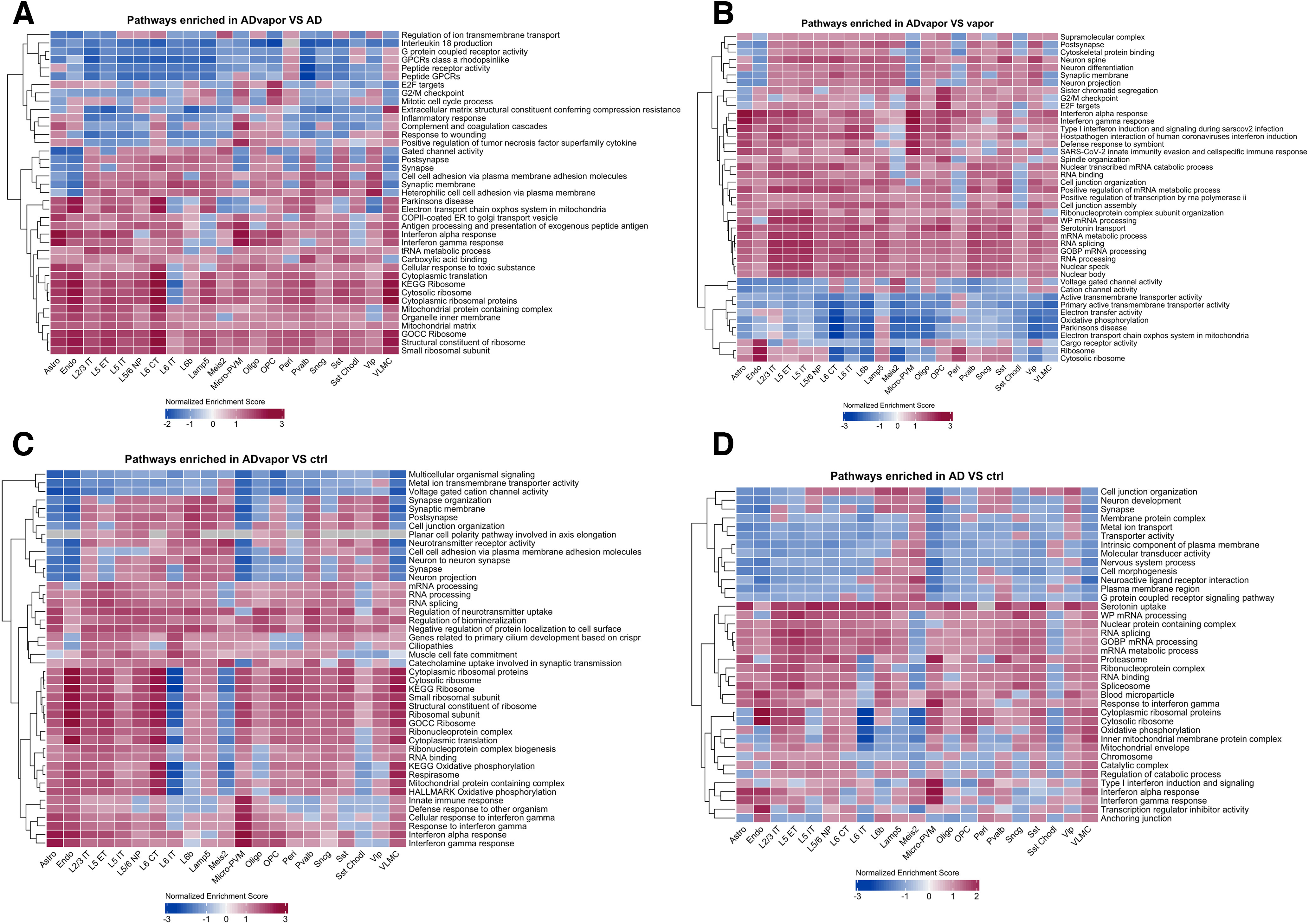
Cell type-specific pathway analysis by GSEA in 3xTg-AD and WT mice with and without a history of repeated alcohol intoxication. ***A–D***, Pathway analysis by GSEA in 3xTg-AD mice with a history of repeated alcohol intoxication by exposure to alcohol vapor (ADvapor) versus 3xTg-AD control mice not exposed to alcohol (AD; ***A***); 3xTg-AD mice with a history of repeated alcohol intoxication (ADvapor) versus WT mice with a history of alcohol intoxication (vapor; ***B***); 3xTg-AD mice with a history of repeated alcohol intoxication (ADvapor) versus WT untreated mice (ctrl; ***C***); and 3xTg-AD mice without alcohol exposure (AD) versus WT mice without alcohol exposure (ctrl; ***D***). For tabular results reporting the significant enriched pathways, see the following: Extended Data Figure 6-1 for panel ***C***; Extended Data Figure 6-2 for panel ***B***; Extended Data Figure 6-3 for panel ***D***; and Extended Data Figure 6-4 for panel ***A***. For the contrast of 3xTg-AD mice with a history of repeated alcohol intoxication versus WT mice without a history of alcohol intoxication but not significant in 3xTg-AD mice without alcohol exposure and WT mice with a history of alcohol intoxication versus ctrl contrasts, see Extended Data Figure 6-5.

**Figure 7.**
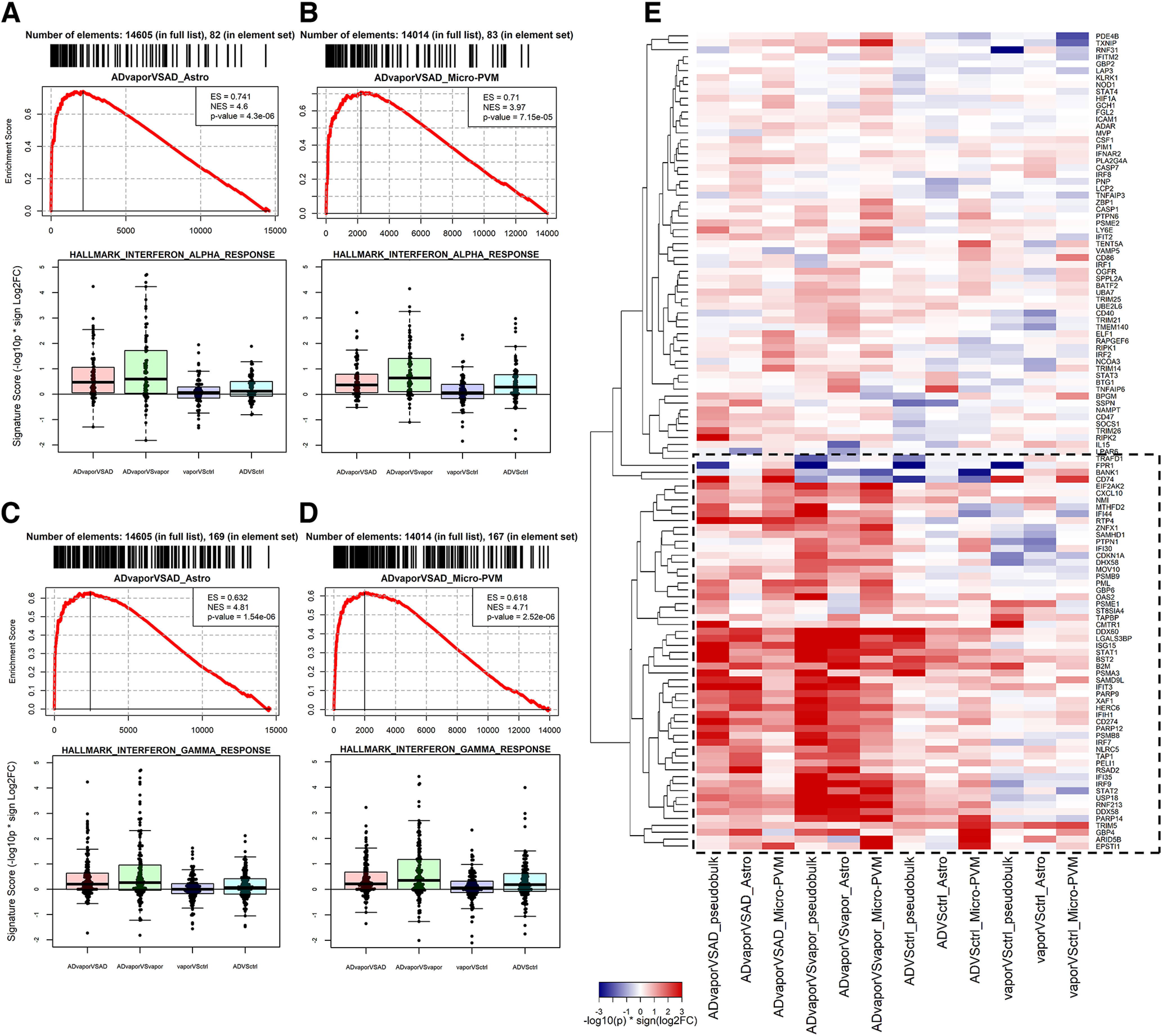
Induction of type I and II IFN in 3xTg-AD mice by a history of repeated alcohol intoxication. ***A***, ***B***, GSEA results for the IFN-α response pathway [the most significantly upregulated pathway in 3xTg-AD mice exposed to alcohol versus 3xTg-AD mice (ADvapor vs AD contrast)] in astrocytes (***A***) and microglia (***B***). ***C***, ***D***, GSEA results for the interferon-γ response pathway (the most significantly upregulated pathway in the ADvapor vs AD contrast) in astrocytes (***C***) and microglia (***D***). Top panels indicate the GSEA analysis in the specific contrast and cell type. Bottom panels use box plots to indicate the differential expression of genes belonging to the relative indicated pathways in the four following contrasts: 3xTg-AD mice exposed to alcohol versus 3xTg-AD mice (ADvapor vs AD; red); 3xTg-AD mice exposed to alcohol versus WT mice exposed to alcohol (ADvapor vs vapor; green); WT mice exposed to alcohol versus WT mice not exposed to alcohol (vapor vs WT ctrl; blue); and 3xTg-AD mice versus WT (AD vs WT ctrl; cyan). ***E***, Heatmap showing differential expression values for genes in IFN-α and IFN-γ response pathways. Genes were selected because of being in the GSEA leading edge in at least one of the following four contrasts: ADvapor versus AD_Astrocytes; ADvapor versus AD_Micro-PVM; ADvapor versus vapor Astrocytes; and ADvapor versus vapor_Micro-PVM. Total: 118 genes. The square defines a subset of IFN-α and IFN-γ pathway genes that show an additive effect in ADvapor.

GSEA revealed biological pathways that were significantly enriched in the four contrasts (Fig. 6, selected nonredundant pathways, Extended Data Figs. 6-1, 6-2, 6-3, 6-4, report of all significantly enriched pathways, Extended Data Fig. 6-5, report of pathways enriched in 3xTg-AD mice exposed to alcohol vs WT mice but not in 3xTg-AD mice or WT mice exposed to alcohol vs WT controls). The expression of gene sets related to type I and II IFN was increased in astrocytes and microglia-PVM of the PFC of 3xTg-AD mice exposed to alcohol versus 3xTg-AD mice and WT mice with and without alcohol exposure (Figs. 6, 7). In astrocytes, gene sets related to calcium signaling were downregulated. In microglia-PVM, pathways related to immune activation and proliferation were upregulated (Fig. 6).

10.1523/ENEURO.0456-22.2023.f6-1 Figure 6-1 Significant (adjusted *p *≤* *0.01) pathways enriched in the contrast of 3xTg-AD mice with a history of repeated alcohol intoxication by exposure to alcohol vapor (ADvapor) versus WT mice not exposed to alcohol (ctrl), according to the *fgsea* algorithm. Each tab represents a specific cell type in which the analysis was performed. Download Figure 6-1, XLS file.

10.1523/ENEURO.0456-22.2023.f6-2 Figure 6-2 Significant (adjusted *p *≤* *0.01) pathways enriched in the contrast of 3xTg-AD mice with a history of repeated alcohol intoxication by exposure to alcohol vapor (ADvapor) versus WT mice exposed to alcohol (vapor), according to the *fgsea* algorithm. Each tab represents a specific cell type in which the analysis was performed. Download Figure 6-2, XLS file.

10.1523/ENEURO.0456-22.2023.f6-3 Figure 6-3 Significant (adjusted *p *≤* *0.01) pathways enriched in the contrast of 3xTg-AD mice without alcohol exposure (AD) versus WT mice without alcohol exposure (ctrl), according to the *fgsea* algorithm. Each tab represents a specific cell type in which the analysis was performed. Download Figure 6-3, XLS file.

10.1523/ENEURO.0456-22.2023.f6-4 Figure 6-4 Significant (adjusted *p *≤* *0.01) pathways enriched in the contrast of 3xTg-AD mice with a history of repeated alcohol intoxication (ADvapor) versus 3xTg-AD mice without a history of alcohol intoxication (AD), according to the *fgsea* algorithm. Each tab represents a specific cell type in which the analysis was performed. Download Figure 6-4, XLS file.

10.1523/ENEURO.0456-22.2023.f6-5 Figure 6-5 Table of pathways significant (adjusted *p *≤* *0.01) in the contrast of 3xTg-AD mice with a history of repeated alcohol intoxication (ADvapor) versus WT mice without a history of alcohol intoxication (ctrl), and not significant in 3xTg-AD mice without alcohol exposure (AD) and WT mice with a history of alcohol intoxication (vapor) versus ctrl contrasts. Download Figure 6-5, XLS file.

Pathways related to synaptic membrane components were selectively upregulated in a cell type-specific manner in neurons of 3xTg-AD mice exposed to alcohol versus 3xTg-AD mice and WT mice with and without alcohol exposure, including in L6 CT, L6b, Lamp5, and parvalbumin, and Meis2 interneurons. Increases in synaptic elements and synaptic and metabolic activity are believed to be compensatory and adaptive mechanisms of the natural history of early AD (Bobkova and Vorobyov, 2015; Merlo et al., 2019).

Gene sets related to ribosomal RNA decreased in a cell type-specific manner in L6 IT neurons of 3xTg-AD mice exposed to alcohol versus 3xTg-AD mice and WT control mice, whereas they increased in most other cell types (Fig. 6*A*,*C*, respectively). A decrease in gene sets related to oxidative phosphorylation, mitochondrial function, and Parkinson-associated genes was seen in Meis2 interneurons, possibly underlying cell type-specific vulnerability (Extended Data Fig. 6-5).

## Discussion

The present study was designed to test the hypothesis that excessive alcohol intake increases AD risk and disease progression and to delineate transcriptional correlates of a history of repeated alcohol intoxication at the single-cell level in a model of AD. We found that a history of repeated alcohol intoxication promotes the emergence of spatial learning and memory impairments in presymptomatic 3xTg-AD mice and transcriptional changes consistent with AD progression in the PFC of 3xTg-AD mice that harbor genetic mutations that underlie early-onset familial AD in humans. The present results indicate that excessive alcohol intake promotes AD progression in the setting of genetic AD susceptibility. These results also show that repeated alcohol intoxication by exposure to alcohol vapor is a robust method to model the interaction between excessive alcohol intake and AD susceptibility genes. The present study also shows that the same level of alcohol exposure selectively induced the emergence of cognitive impairment in 3xTg-AD and 5xFAD mice, whereas their respective WT littermate controls were unaffected. Consistent with our findings, Barnett et al. (2022) recently reported that repeated binge alcohol administration by gavage in adolescent 3xTg-AD mice promotes cognitive impairment later in life, and Hoffman et al. (2019) showed that a 4 month history of 24 h, two-bottle choice home-cage consumption of saccharin-sweetened alcohol also impaired spatial memory in 3xTg-AD mice. It is important to note that the murine models used in the present study and in those previous studies have significant limitations that include discrepancies in the pathologic presentation, high levels of transgene expression, accelerated disease progression, and a nonphysiological combination of familial AD mutations, among others (Kitazawa et al., 2012).

The present single-cell level gene expression dataset provides a powerful resource for deconvolving gene regulatory relationships and identifying candidate therapeutic targets for AD. To begin to delineate mechanisms of the detrimental interaction between AD susceptibility and excessive alcohol intake, we performed gene expression profiling at single-cell resolution. We identified gene signatures and pathways that are regulated in a cell type-specific manner by a history of repeated alcohol intoxication in 3xTg-AD mice. Several differentially expressed genes support previously recognized and potentially new candidate mechanisms of AD pathogenesis. Importantly, only a few of these genes that were differentially expressed in specific cell types also showed differential expression in pseudo-bulk signatures, which supports the value of the added resolution of the single-cell approach.

Deep and superficial excitatory neurons showed a broad increase in the transcription of cadherins that are upregulated in seizures and implicated in sprouting, synapse formation, and excitatory/inhibitory balance (Shan et al., 2002; Williams et al., 2011; Smith et al., 2017); therefore, they may also underlie increased excitability in the cortex in AD and a history of excessive alcohol exposure. Changes in the expression of the glycine receptor Glra2 and glutamate receptor GluRδ2 may also be related to the increased excitability of cortical circuits. We observed the increased expression of Slc1a3, the EAAT1, in 3xTg-AD mice exposed to alcohol, which appears consistent with the increased excitatory/inhibitory balance in AD patients, leading to cortical and hippocampal hyperexcitability and cognitive impairment (Palop et al., 2007; Lauterborn et al., 2021; Zhang et al., 2022). We also found that several human AD-associated genes identified by GWASs are significantly differentially regulated by alcohol in specific cell types of both superficial and deep layers of the cortex in 3xTg-AD mice.

Among the genes with increased expression in astrocytes of 3xTg-AD mice with a history of repeated alcohol intoxication are genes previously implicated in AD pathogenesis, such as complement C4b, implicated in neuroinflammation in AD and other conditions (Jun et al., 2022); the IFN-regulated gene Ifit3 (Hur et al., 2020); the antioxidant and heavy metal binding protein Mt1 that is upregulated by neuroinflammation and was previously found to be upregulated in AD and AD models (Carrasco et al., 2006; Manso et al., 2016); and cathepsin S, a likely contributor to neuroinflammation in AD (Lowry and Klegeris, 2018). The downregulation of Atg16l2, a negative regulator of the NLRP3 inflammasome (Wang et al., 2022), may contribute to neuroinflammation. We also found transcriptional evidence of type I and II interferon activation. Type I IFN was recently shown to be induced in 3xTg-AD mice, also by repeated binge alcohol administration by gavage (Barnett et al., 2022). Evidence from animal models suggests that IFN signaling contributes to cognitive symptoms and disease progression (Minter et al., 2016), and IFN activation has also been demonstrated in human AD plaques (Roy et al., 2020). While Tlr7 increased selectively in 3xTg-AD mice exposed to alcohol, it increased in the brains of humans with alcohol use disorder (Coleman et al., 2017) and in genes related to antigen presentation, such as H2-K1 and H2-D1, among others. Upregulated genes in endothelial cells of 3xTg-AD mice exposed to alcohol were indicative of aged capillaries, including innate immunity genes, such as Ly6a, Ly6c1, Ifitm3, B2m, H2-K1, and H2-D1 (Chen et al., 2020).

Ribosomal proteins were broadly dysregulated by a history of repeated alcohol intoxication in 3xTg-AD mice. Changes in ribosomal content and function are increasingly recognized as pathogenic mechanisms, including in neurodevelopmental and neurodegenerative disorders (Farley-Barnes et al., 2019), aging (Zahn et al., 2006, 2007; Ximerakis et al., 2019), and cellular senescence (Nishimura et al., 2015). An additional indicator of altered proteostasis is the differential expression of cyclin-dependent kinase Cdk8, a regulator of transcription (Dannappel et al., 2018) that is also affected by alcohol in the liver (Song et al., 2017). We found an unexpected broad increase in synapse-related transcripts, which may reflect early stages of AD in which compensatory and adaptive mechanisms, including increases in synaptic proteins, predominate (Bobkova and Vorobyov, 2015; Merlo et al., 2019). Several markers of aging in endothelial cells (Chen et al., 2020) were selectively upregulated in 3xTg-AD mice exposed to alcohol.

Comparisons of gene expression signatures of 3xTg-AD mice with and without histories of repeated alcohol intoxication versus WT control mice to signatures of 3xTg-AD mice and 5xFAD mice of different ages showed that transcriptomes of 3xTg-AD mice with histories of repeated alcohol intoxication had greater similarities to the transcriptomes of older 3xTg-AD mice and 5xFAD mice with advanced disease and cognitive impairment than the transcriptomes of 3xTg-AD mice not exposed to alcohol, indicating that alcohol promotes accelerated AD progression.

Overall, the present study provides a comprehensive gene expression dataset at the single-nucleus level of the interaction between a history of alcohol intoxication in the setting of genetic AD susceptibility in mutant mice, which results in cognitive impairment. Our data highlight microglia and astrocyte neuroinflammatory mechanisms in the action of alcohol in AD pathogenesis and support a role for alcohol in promoting neuroinflammation and disease progression in prodromal and early AD. To the best of our knowledge, the present resource is the first single-cell view of transcriptional alterations brought about by an AD–alcohol interaction, revealing cell type-specific and shared gene expression perturbations. There is an urgent need for effective therapeutics to reverse or at least delay AD progression. The present single-cell level gene expression dataset provides a powerful resource for deconvolving gene regulatory relationships and identifying candidate therapeutic targets for AD progression and the role of excessive alcohol intake in cognitive impairment.

## Synthesis

Reviewing Editor: Leandro Vendruscolo, National Institute on Drug Abuse Decisions are customarily a result of the Reviewing Editor and the peer reviewers coming together and discussing their recommendations until a consensus is reached. When revisions are invited, a fact-based synthesis statement explaining their decision and outlining what is needed to prepare a revision will be listed below. The following reviewer(s) agreed to reveal their identity: NONE.

N/A
