# Supplementary figures and images for: A History of Repeated Alcohol Intoxication Promotes Cognitive Impairment and Gene Expression Signatures of Disease Progression in the 3xTg Mouse Model of Alzheimer’s Disease
Source: eNeuro. 2023 Jul 4;10(7):ENEURO.0456-22.2023. doi: 10.1523/ENEURO.0456-22.2023 (PMC10337838; doi:10.1523/ENEURO.0456-22.2023)

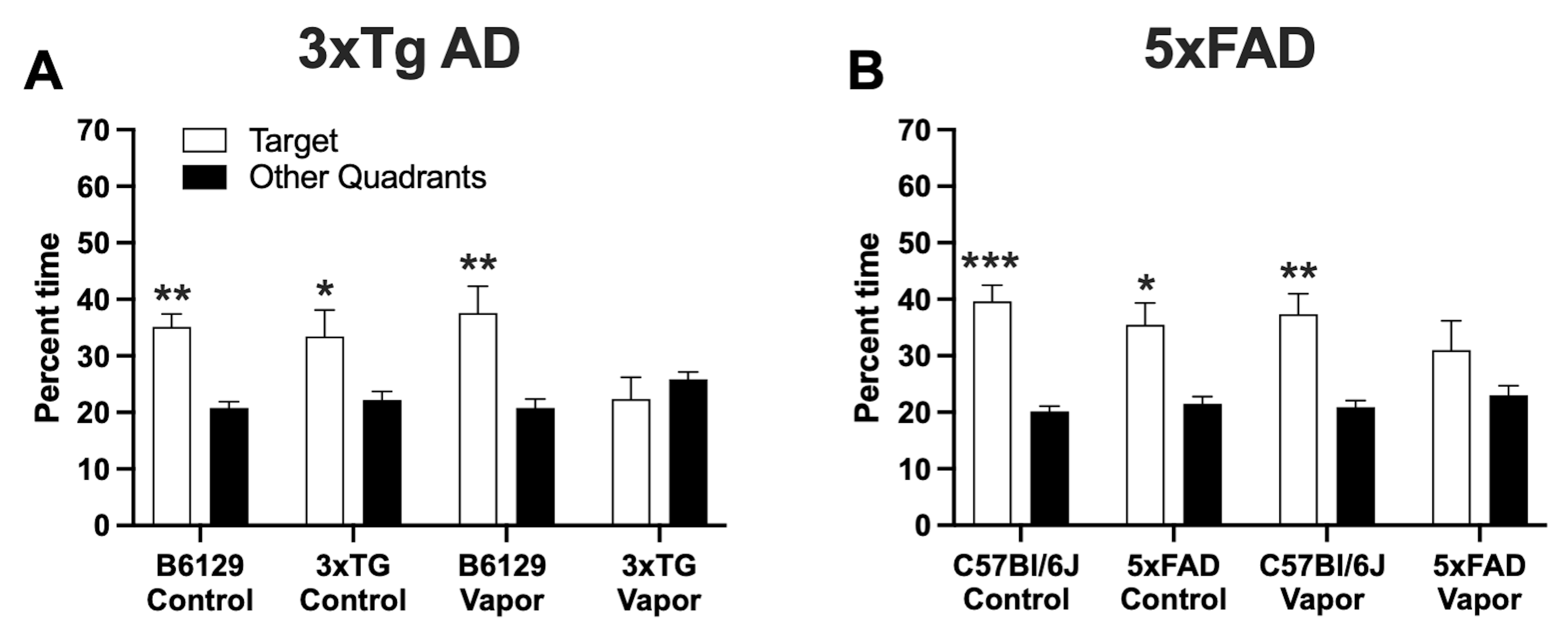

Supplement: Figure 1-1 — Repeated alcohol intoxication by alcohol vapor exposure hastens the onset of cognitive impairment in 3xTg-AD and 5xFAD mice in the Morris water maze (MWM). (A) A separate cohort of male 3xTg-AD mice showed impaired spatial memory after five cycles of chronic intermittent alcohol vapor in the MWM, in which they spent comparable time in the target quadrant where the hidden platform was located as in the other quadrants of the pool (3xTG Vapor). Conversely, 3xTg-AD mice not exposed to alcohol (3xTG Control) and WT mice either exposed or not exposed to alcohol vapor spent significantly more time in the target quadrant compared to the other quadrants (B6129 Control and B6129 Vapor). Three-way analysis of variance revealed a significant main effect of quadrants (F1,52 = 11.64, p < 0.01) and a quadrant genotype interaction (F1,52 = 4.201, p < 0.05). The data represent the mean ± SEM. n = 7-20. *p < 0.05, **p < 0.01, Fisher's Least Significant Difference test. (B) Similarly, male 5xFAD mice also showed impaired spatial memory after three cycles of chronic intermittent alcohol vapor in the MWM (5xFAD Vapor), whereas 5xFAD mice not exposed to alcohol (5xFAD Control) and WT mice either exposed or not exposed to alcohol vapor (C57Bl6/J Control and C57Bl6/J Vapor) spent significantly more time in the target quadrant compared to the other quadrants. Three-way analysis of variance revealed a significant main effect of genotype (F1,52 = 28.75, p< 0.0001). The data represent the mean ± SEM. n = 10-20. *p < 0.05, **p < 0.01, ***p < 0.001, Fisher's Least Significant Difference test. BALs are shown in Fig 1-2. Download Figure 1-1, TIFF file. [file enu-eN-MNT-0456-22-s03.tiff]

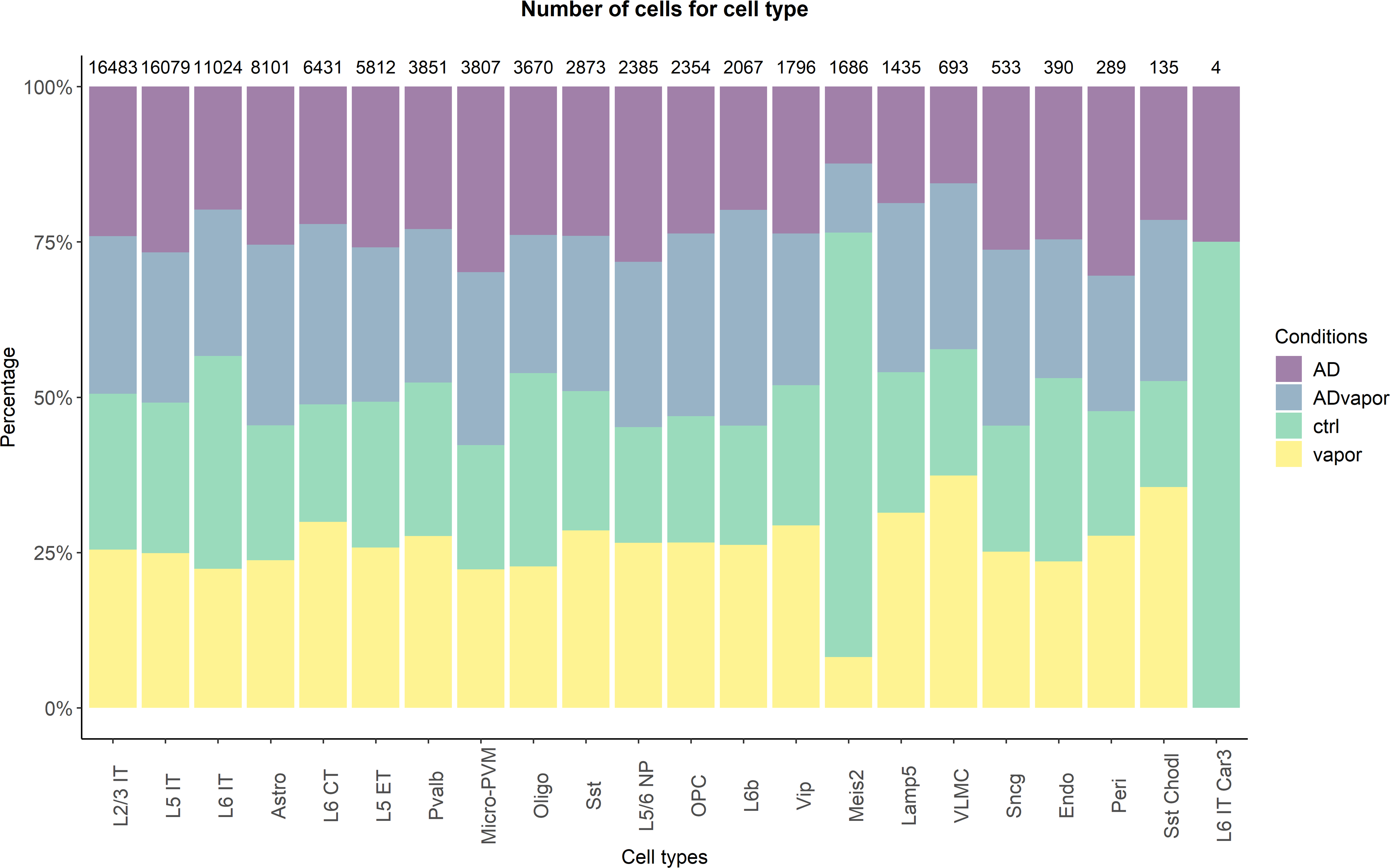

Supplement: Figure 2-2 — Stacked bar plot showing the distribution of cell types in the four experimental conditions. Total number of cells in the dataset are shown at the top of each bar. Download Figure 2-2, TIF file. [file enu-eN-MNT-0456-22-s05.tif]

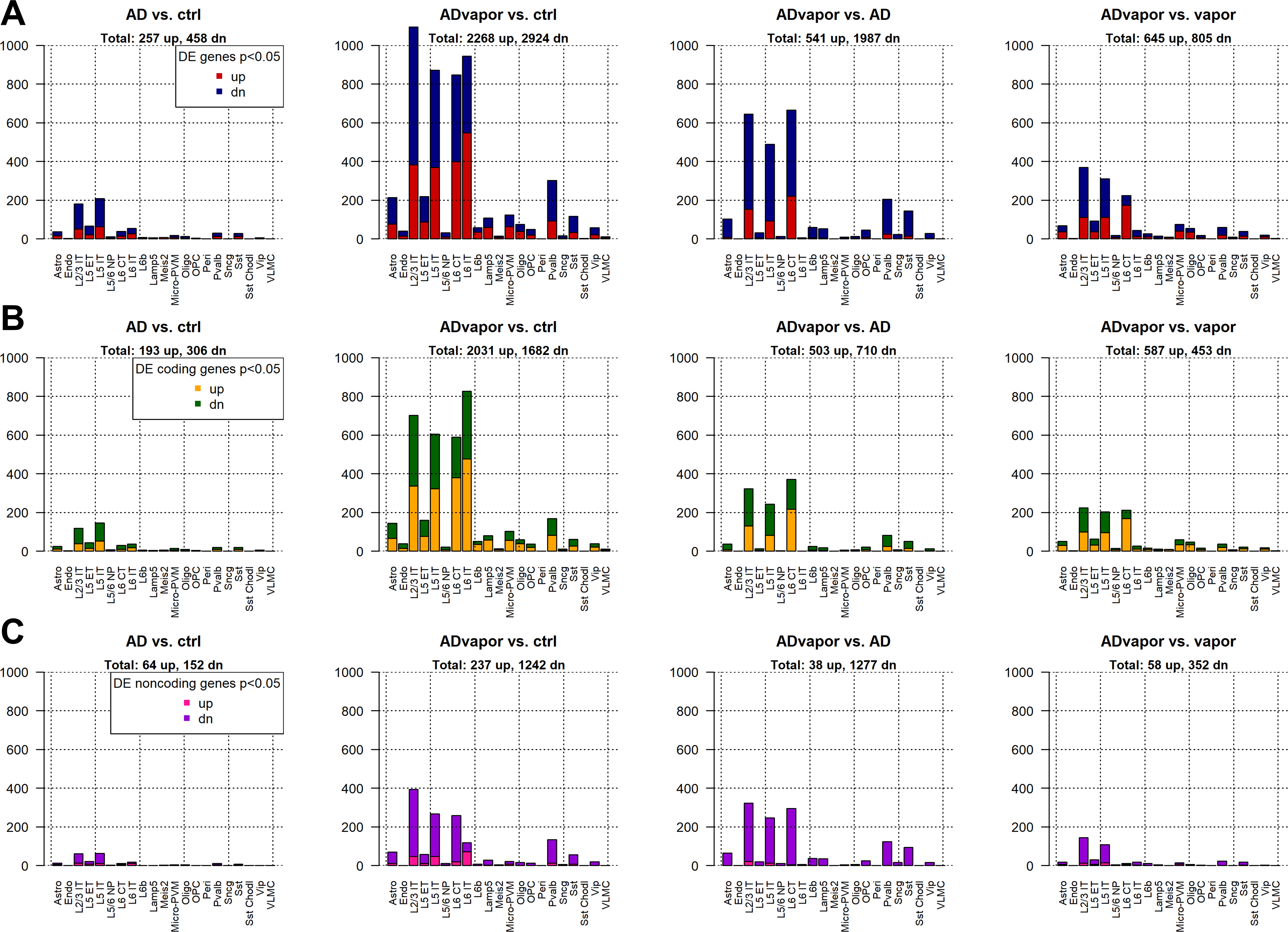

Supplement: Figure 3-1 — Distribution of differentially expressed genes in each cell type in the four contrasts. A, Differential expression analysis in four contrasts, representing the total number of genes differentially expressed at an adjusted p ≤ 0.05, divided into upregulated (up) and downregulated (dn). B, As in A, with numbers referring to protein-coding genes only. C, As in A, with numbers referring to noncoding genes. AD, 3xTg-AD mice; Advapor, 3xTg-AD mice with a history of repeated alcohol intoxication by alcohol vapor exposure; ctrl, WT control mice not exposed to alcohol; vapor, WT control mice with a history of repeated alcohol intoxication by alcohol vapor exposure. Download Figure 3-1, TIF file. [file enu-eN-MNT-0456-22-s06.tif]
