# Supplementary material for: A History of Repeated Alcohol Intoxication Promotes Cognitive Impairment and Gene Expression Signatures of Disease Progression in the 3xTg Mouse Model of Alzheimer’s Disease
Source: eNeuro. 2023 Jul 4;10(7):ENEURO.0456-22.2023. doi: 10.1523/ENEURO.0456-22.2023 (PMC10337838; doi:10.1523/ENEURO.0456-22.2023)
Supplement: Figure 2-1 — Abbreviations for cell types. Download Figure 2-1, DOCX file. [file enu-eN-MNT-0456-22-s04.docx]

**Figure 2-1. Abbreviations**

| **Acronym** | **Expanded cell type name** |
| --- | --- |
| Astro | Astrocyte |
| Endo | Endothelial cell |
| L2/3 IT | Layer 2-3 glutamatergic neuron, intratelencephalon-projecting |
| L5 ET | Layer 5 glutamatergic neuron, extratelencephalon-projecting |
| L5 IT | Layer 5 glutamatergic neuron, intratelencephalon-projecting |
| L5/6 NP | Layer 5-6 glutamatergic neuron, near-projecting |
| L6 CT | Layer 6 glutamatergic neuron, corticothalamic-projecting |
| L6 IT | Layer 6 glutamatergic neuron, intratelencephalon-projecting |
| L6 IT Car3 | Layer 6 Car3+ glutamatergic neuron, intratelencephalon-projecting |
| L6b | Layer 6b glutamatergic neuron |
| Lamp5 | Lamp5+ GABAergic neuron |
| Meis2 | Meis2+ GABAergic neuron |
| Micro-PVM | Microglia / perivascular macrophage |
| Oligo | Oligodendrocyte |
| OPC | Oligodendrocyte precursor cell |
| Peri | Pericyte |
| Pvalb | Parvalbumin+ GABAergic neuron |
| Sncg | Gamma-synuclein+ GABAergic neuron |
| Sst | Somatostatin+ GABAergic neuron |
| Sst Chodl | Somatostatin+ chondrolectin+ GABAergic neuron |
| Vip | Vasointestinal peptide+ GABAergic neuron |
| VLMC | Vascular leptomeningeal cell |
